# Progress in Surgery

**Published:** 1896-05-30

**Authors:** 


### Progress in Surgery.

### BRAIN SURGERY.

It is a hopeful and encouraging sign of the times to find *physicians* writing "on the results of *surgical* measures in a series of cases of so-and-so." Various contributions of this kind have recently appeared, and although all have not been favourable to the continued application of surgical means in the conditions of which they treat, the opinions of the medical writer are none the less welcome and valuable to his surgical *confrère*. Dr. G. A. Gibson,[1] of Edinburgh, gives his experience of surgical treatment in cerebral cases in a paper which he concludes thus: "To sum up is an easy task, as it is almost entirely comprised in the advice to operate early, which can only be rendered possible by the loyal co-operation of the physician and surgeon. We sometimes hear the statement that the diagnosis of such cases belongs solely to the physician, and that when he has made up his mind he has simply to issue instructions to the surgeon. In fact, that the surgeon is simply the hand, the physician the head. This, however, is a point of view that should be warmly repudiated. Not only does it throw discredit upon a great branch of our profession, but it also renders the mutual helpfulness of medicine and surgery impracticable, and prevents the full benefits which accrue from the harmonious co-operation of real fellow-workers." The paper refers to seven cases in which brain lesions have been treated by operative measures. The clinical features are very fully described. In cases (1) and (2) new growths were successfully removed by Mr. Annandale, and the patients completely recovered. Of these one was a gliosarcoma of the motor cortex, and the other a fibrosarcoma of the cerebellum. (3) In another case a glioma was not found at the operation, but post-mortem was localised in the corpus striatum. (4) A case of infantile hemiplegia with convulsions was little the better for the operation, although the writer says that "with similar conditions he should follow the same course as on this occasion." (5) A weak-minded woman of forty-seven had convulsive attacks which were believed to be due to a focus of cortical irritation. An exploratory operation was performed with negative results. The patient died, and the brain was found to exhibit all the evidences of general paralysis of the insane. (6) Severe pain, which was attributed to an old fracture of the skull, was relieved by trephining and removing an area of bone, including a depression. (7) The last case was one of compression from recent injury in which operation failed to do good.

**Optic Neuritis in Brain Lesions.**—In the discussion on Dr. Gibson's paper, Dr. Argyl Robertson,[2] in reply to the question how long, and to what extent, optic neuritis might exist, and yet after removal of intra-cranial tension by trephining return to the normal, said that he had seen several cases of very acute thoroughly developed optic neuritis in patients suffering either from cerebral tumours or injuries, in whom, after operation, very good, almost perfect vision had been restored in some instances. Whether perfect vision is restored or not depends upon the length of time the neuritis has existed, and whether or not complete atrophy of the optic nerve fibres has taken place. Recovery may take place from anything short of complete atrophy. It is well known that there may be extensive optic neuritis without much interference with vision, because the impression formed on the retina is perfect, is conveyed to the brain, and accurately perceived by the patient. The difficult point to determine is when atrophic changes ensue in the nerve elements. It depends largely on the extent of the neuritis. In mild cases it is slow, and in acute cases more rapid.

**A New Symptom Localising Cerebellar Lesions.**—Dr. Purves-Stewart,[3] of Edinburgh, calls attention to a symptom which he thinks may prove of value in the localisation of cerebellar lesions. He mentions that all the localising symptoms have hitherto been associated only with the middle lobe—which is the essential part—of the cerebellum. The symptom he refers to, viz., fronto-cerebellar headache, is associated with lesions in the lateral lobes, and by its aid a tumour was successfully localised in one of Dr. Gibson's cases above referred to.[1] The tumour was on the right side, and the pain was in the left frontal region. The pain is explained by pressure on those fibres of the superior peduncle (the fronto-cerebellar tract) which, after decussating between the corpora quadrigemina, pass to the frontal lobe of the opposite cerebral hemisphere. This symptom has been found useful in a second case.

**Surgical Exploration of the Brain.**—Dr. Doyen,[4] in a recent paper, advocates very free exposure of the brain in dealing with intra-cranial conditions. Cerebral localisation he looks upon as chiefly of physiological interest, all that the surgeon requires being merely an indication of the side on which a lesion is. The brain he places in the same category as other viscera for surgical purposes, and he exposes it with like freedom. For exploratory purposes he exposes bone from forehead to occiput, and with the aid of an electro-motor trephine going at the rate of 3,000 turns a minute, removes several circles at intervals from one another. They are then united by a circular saw and a large osteo-cutaneous flap thrown down. The dura is reflected as a flap, if necessary, and replaced and fixed in position afterwards. Such an operation he considers no more dangerous than a laparotomy.

**Craniectomy for Microcephalus.**—Drs. Shaw and Dendy[5] report another unsuccessful operation for this condition. The operation was done in two stages, death following the second operation. A large subdural hæmatoma was found excavating the left hemisphere. Dana[6] gives the results in 81 cases where this operation has been done. Of these 35 were improved, 22 not improved, and 24 deaths. The writer thinks the operation indicated in congenital idiots and imbeciles. Where cerebral diplegia is associated with mental defects the operation should not be done. The children become more tractable and cleanly, and their speaking and swallowing are improved. Linear craniotomy with lateral branches he considers the best operation. He considers that the operation produces good effects, "because of its profoundly disciplinary effect upon the idiot."

**Surgical Treatment of Hydrocephalus.**—Félix Ferrier[7] indicates the three methods by which hydrocephalus may be treated surgically. (*a*) Compression has had its trial, and has failed. (*b*) Evacuation, if it does not always cure, at least gives temporary relief. The puncture may be into the lateral ventricle directly through a small trephine opening, and the fluid slowly withdrawn. Sometimes a horsehair drain is kept in for a time, and sometimes fluid—sterilised water, boracic lotion, or other medicated solution—is injected. In some cases the puncture is into the sub-arachnoid space only, preferably near the base, so that the ventricles are drained through the foramen of Majendie, if this opening be not closed by adhesions. (*c*) Lumbar puncture has been employed more recently with varying results.

**Bone Grafting for Cranial Defect.**—In two cases where portions of the cranial vault had been destroyed Dr. Eastman,[7] of Burlington, has filled up the gap by an ingenious method. A flap of soft tissue having been reflected, and the defect in the bone thoroughly exposed, the dura mater was loosened from the edge. A pattern of the gap was next made in sterilised rubber tissue, and placed on an adjacent portion of healthy bone. With this as a guide the bone is cut through as far as the diploe, and the outer table prised off. The graft thus obtained is placed in the gap, the soft parts restored to their position, a capillary drain established, and the wound closed. There is sharp bleeding at the operation, but little shock, and, with asepsis, primary union. The results have been most satisfactory, and undoubtedly better than any artificial plate of silver, celluloid, or rubber could give.

[1] Edin. Med. Jour., Feb., 1896. [2] Ibid., Jan., 1896. [3] Presse Médicale, Nov. 6, 1895. [4] Pediatrics, 1896. [5] Lancet, Dec. 7, 1895, [6] Jour. de Chir. et Thér. Infantiles, 43, 1895. [7] Ann. Surg., Dec., 1895.

### HEAD INJURIES.

**Cerebral Concussion.**—A contribution to the theory of cerebral concussion has recently been made by Dr. S. P. Kramer,[1] of Cincinnati. There is no constant gross lesion which will satisfactorily account for the diffuse disturbance of the nervous system in this condition. The old idea that the symptoms are due to a molecular shaking up of the brain tissue is no longer tenable. The writer objects to the theory of Duret, which attributes the symptoms to increased tension of the cerebro-spinal fluid in the ventricles of the brain, especially the fourth, on the following grounds—(1) That it does not explain the injuries to the rest of the brain, as evidenced by the cortical paralysis or coma; (2) that injury to the fourth ventricle will not produce coma; (3) that concussion can be produced in animals after draining off the cerebro-spinal fluid through an opening in the occipito-atlantoid membrane; (4) that physically it is impossible for the intra-ventricular ever to be greater than the extra-ventricular tension. He considers the explanation suggested by Stromeyer more rational, that the blow by depressing the skull produces a momentary increase in the intra-cranial pressure, which compresses the blood-vessels, and so cuts off the supply of blood. Kramer's own experiments on dogs subjected to blows of varying intensity gave the following results: (1) Respiratory changes.—The immediate effect of the blow was in general one which had a paralysing effect upon the respiratory centres in the medulla. This was shown by the immediate cessation of respiration on receipt of the blow, the thorax remaining in the position of expiration. This was temporary or permanent, according to the severity of the blow. (2) Circulatory changes.—In severe concussions the blow was immediately followed by a slight fall in blood pressure; there was no appreciable change in cardiac rhythm, and the force of the heart's action increased appreciably after the blow. (3) Intra-cranial tension was always suddenly increased and then rapidly fell to normal when the depression in the skull righted itself. He concludes that (*a*) a blow to the head produces a momentary increase of intra-cranial tension and consequent compression of the brain as a whole; (*b*) the effect of the compression is to cause an interference with the blood supply of the entire brain, and this is sufficient to account for the primary symptoms of cerebral concussion; (*c*) the so-called syncopic death after severe concussion is produced by a paralysis of the respiratory centres, the cardiac centres remaining intact. The fatal result may in many cases be prevented by the prompt institution of artificial respiration.

**Head Injury with Aphasia.**—An interesting case is reported by Dr. E. H. Williams,[2] of a man, nineteen, who was hit by a stone on the left side of his head. He was rendered unconscious, and had immediately a severe convulsive attack lasting nine minutes. He improved later, but was found to suffer from amnesic aphasia, which persisted for some weeks, gradually passing off. During this time he had several epileptiform fits, and had loss of power in the opposite arm. The diagnosis was easy, but no operation was performed, in spite of a well-marked depression at the seat of the blow. The patient recovered.

**Traumatic Cephalhydrocele.**—Mr. Hogarth Pringle[3] reports two cases of this rare condition in the "Edinburgh Hospital Reports." One, a man aged twenty, when sixteen months old, fell a distance of fourteen feet, sustaining injury to the left side of the head. He remained dull and stupid, had fits at varying intervals, and a pulsating tumour remained on his head. At the request of the parents, Mr. Annandale operated in the hope of improving the mental condition. On dissecting back a flap, a large lax-walled cyst was met with, containing a large quantity of clear yellowish fluid. An aperture in the skull admitted the little finger. The patient did not survive, and at the post-mortem examination it was found that a channel led from the cyst through the cerebral hemisphere into the lateral ventricle. The second case was a little girl of four, who three years previously had fallen down a stair. She was found unconscious, with a swelling above and in front of the auricle. An incision gave vent to dark-coloured blood, and the swelling disappeared. It gradually returned, however, and became pulsatile. There was no mental impairment, nor fits. Some fluid withdrawn by a hypodermic needle proved to be cerebro-spinal fluid. The swelling diminished in size, lost its pulsation, but did not entirely disappear. The writer is of opinion that the opening in the bone which was present in both of these cases depends on absorption of bone devitalised at the time of the injury.

**Traumatic Epilepsy.**—M. Allen Starr[4] states his present opinion on the value of operative treatment of traumatic epilepsy in a paper read before the New York State Medical Society. The only type of epilepsy in which the question of operation can arise is that of so-called cortical epilepsy, where an irritant lesion of one region of the brain produces localised spasms. Such lesions may be traumatic in origin from fractures, localised meningitis, small hæmorrhages or cysts; or the epilepsy may be the first sign of a beginning tumour, abscess, or sclerotic patch. It is for this class of cases that operations have been largely undertaken in the past, and are still being done. The writer goes on to say that he is exceedingly disappointed with the final results of operations in this type of disease. Experience seems to show that, although after an operation a cessation of attacks may occur, these attacks are almost certain in the end to recur, and hence the patients are not actually cured. He is becoming more and more reluctant to recommend operation. In cases of epilepsy due to a cicatrix in the brain substance there is little to be hoped for from operation, because a similar scar will result from the excision of the primary one, and little permanent good result.

An interesting case of traumatic epilepsy is reported by W. Turner, of Gibraltar.[4] The patient, aged 39, complained of partial motor paralysis and numbness in the left half of the body, face, and in his left limbs, headache, drowsiness, defective eyesight and memory, and impairment of speech, with occasional epileptiform convulsions. Four years previously he had sustained a depressed fracture of the vertex of the skull, followed by severe spasmodic seizures. These convulsions recurred at intervals and his other symptoms ensued. When admitted to the Colonial Hospital he had a deep depressed fracture across the vertex of his skull, and a sinus discharging near the inferior angle of the right parietal bone. On trephining the skull it was found that this sinus led down to a large extra-dural abscess cavity containing sero-purulent material and a sequestrum of the inner table. This was drained, and the patient made a rapid and complete recovery. Dr. J. D. Davis[5] reports a somewhat similar case in which the removal of some softened bone was followed by cessation of epileptiform fits and other head symptoms. A second case was improved by evacuating a small sub-dural abscess; and a third was cured by trephining, although no gross lesion was found to explain the great increase in intra-cranial tension.

Mr. A. E. Morrison[6] reports a case in which the exciting lesion was a large sub-arachnoid cyst following on an injury. This was opened and emptied, and the patient recovered completely.

**Traumatic Insanity.**—Two successful cases of operation for traumatic insanity are recorded by G. W. Cale, of St. Louis.[7] The first was a man, twenty-six, who in 1885 received a blow over the parietal bone on the left side. He subsequently required to be detained in a lunatic asylum. In 1888 Dr. Cale operated, and the patient recovered. The second was a case of acute mania, following an injury. Fuller[7] and Frank[7] have each operated for traumatic insanity, with but partial success.

**Fracture of Ethmoid with Basal Meningitis.**—An interesting case is reported by W. H. Brown[8] in which a boy was struck by a stone on the occiput and fell forward on his forehead. Three days later a profuse watery discharge (believed to be cerebro-spinal fluid) escaped from both nostrils, and continued for six days. Its cessation was followed by a rise of temperature and other signs of basal meningitis, from which the patient suffered severely but ultimately recovered.

[1] Annals of Surg., Feb., 1896. [2] N. Y. Med. Record, Dec., 1895. [3] Edin. Hosp. Rep., vol. iii. 1895. [4] Lancet, Jan. 11, 1896. [5] Philadelphia Med. News, 1895. [6] B.M.J., Nov. 30, 1895. [7] N. Y. Med. Jour., Jan. 4, 1896. [8] Lancet, Dec. 14, 1895.

### DISEASES OF THE UPPER AIR PASSAGES.

(*Continued from page* 128.)

**Nasal Obstruction.**—Why is some degree of organic obstruction in the nasal passages so common? Mayo Collier[29] has endeavoured to reply to this important question—important, because in recognising the true cause we find the best guide to prevent the occurrence of the deformity. Out of 1,050 patients indiscriminately examined by him at the North-West London Hospital only 110 had normal noses, that is to say, fairly vertical septa and symmetrical nasal cavities. The ages varied from one year to eighty, but it was extremely rare to find any obstruction, unless due to temporary causes, below ten years. Further, in young persons before puberty we may expect to find a majority of normal septa. From these facts we may infer that our nose troubles are not born with us, but are cultivated as we advance in years. In the College of Surgeons there are 2,152 dried skulls, and of these 1,657, or 76 9 per cent., have deflections or other irregularities of the nasal cavities. But both Sir Morell Mackenzie and Zuckerkandl found that about eighty per cent. of aborigines' nasal fossæ were normal, and H. Allen, in ninety-three negroes' skulls found deflections or irregularities in only 21·5 per cent. Collier reminds us that the living nose is a valve, which without living structures to regulate the opening would allow the passage of air in expiration only, and that proof of this is furnished in deep chloroform or other narcosis, some cases of hemiplegia, lesions of the facial nerves, and other states, and he recalls the fact that the position of the nostrils differs as does the shape of the nose in infancy and adult life. He contends that frequently recurring temporary nasal obstruction in the young causes a permanent falling in of the septum towards the most blocked side, from the mechanical effect of rarefaction of the air due to forcible inspiration through the blocked passage. If a bent piece of glass with mercury in the bend be connected by a fairly thick elastic tube and inserted into the nostril during every inspiration the mercury will fall in one limb and rise in the other to the extent of about one inch. Thus the stream of air passing out of the nasal cavity into the lungs exhausts the air in the closed nasal cavity to the extent of one inch of mercury, more or less. If the nostril be blocked the pressure of one inch of mercury is therefore exerted on the wall of the blocked nostril at each inspiration. Now, as the weight of the atmosphere equals about twenty-nine inches of mercury (at sea-level) and exerts a pressure of something like 15 lb. on every square inch, one inch of mercury pressure is equal to about half a pound to the square inch. As the area of the septum equals about nine square inches, we see that the comparatively large force of 4½ lb. may be exerting itself at every inspiration, not only in the thin septum, but on every side of the nasal fossa. Hence the pinched and approximated upper maxillary bones in cases of long-standing nasal obstruction; the high-arched palate and irregular dental arch with crowded teeth; the lessened pharyngeal and post-nasal space; and the tendency to breathe entirely by the mouth, and many other attendant consequences. During sleep more damage is done than during consciousness. Mayo Collier does not entirely exclude other causes of nasal septal deformities, and refers to these under the following heads: (1) Congenital causes; (2) syphilis; (3) rickets; (4) traumatisms; (5) injury to facial nerve; (6) swellings, growths or foreign bodies in nose. The other causes which Collier does not endorse, but which have been advanced to explain degrees of chronic nasal obstruction at one time or another, are: (7) habitually blowing the nose with one hand; (8) habitually sleeping on one side; (9) tendency to vertical overgrowth of the septum; (10) primary laws of organization at fault; (11) action of astringents; (12) habit of putting the finger in the nose; (13) overgrowth of the component parts of the septum.

Finally, Collier cites Ziem's results from artificially blocking one nostril in young animals, which produced deviation of the intermaxillary bone towards the shut up side lesser length of the nasal bone, of the frontal bone, and of the horizontal plate of the palate bone, etc., etc.

**Ozœna** is one of the most intractable diseases known, and Stoker's[30] treatment, which has afforded such excellent results, should be widely known. The method consists in the inhalation of oxygen at frequent intervals, say every second hour during the day, the only other treatment being the use of warm water to syringe out the nose two or three times a day. His method is to fill a bag which contains one cubic foot of oxygen; the bag is placed between boards to obtain pressure, and then by means of a tube with a tap (which empties the bag) carrying an india-rubber nose piece the oxygen is inhaled. If the treatment is continued longer than one hour at a time the author finds that headache is induced. In a case recorded the treatment was continued one month, and the result was highly successful. He believes that one ought to combine the use of usual alkaline and antiseptic lotions, but hitherto he has avoided these in order to demonstrate that the oxygen alone has the beneficial effect. Stoker has employed the oxygen treatment for various other suppurative diseases of the ears and for septic ulcers with equally good results.

[29] Journ. of Laryng., Mar., 1896. [30] Journ. of Laryng. Mar., 1896.

